# Is Achieving the Guidelines of Four Forms of Physical Activity Associated with Less Self-Reported Health Complaints? Cross-Sectional Study of Undergraduates at the University of Turku, Finland

**DOI:** 10.3390/ijerph17155595

**Published:** 2020-08-03

**Authors:** Walid El Ansari, Abdul Salam

**Affiliations:** 1 Department of Surgery, Hamad General Hospital, Doha 3050, Qatar; 2 College of Medicine, Qatar University, Doha 3050, Qatar; 3 School of Health and Education, University of Skovde, 54128 Skövde, Sweden; 4 Department of Epidemiology and Biostatistics, King Fahad Specialist Hospital, Dammam 31444, Saudi Arabia; abdul.or.salam@gmail.com

**Keywords:** self-reported symptoms/health complaints, physical activity, Finland, university students, psychosomatic symptoms

## Abstract

Very little research has assessed the physical activity (PA) of university students in in Finland, and their associations with self-reported health complaints (HCs), whilst simultaneously accounting for a range of other potential confounders. Students at the University of Turku (1177) completed an online health and wellbeing questionnaire that assessed 22 physical and somatic HCs, and students’ achievement of the international guidelines of four forms of PA (moderate, vigorous, moderate-to-vigorous and muscle strengthening PA; MPA, VPA, MVPA, MSPA respectively). We also explored the associations between HCs and PA, controlling for sociodemographic and health confounders (age, sex, year of study, marital status, accommodation during semesters, health awareness). Factor analysis reduced the HCs into three factors (psychological, pains/aches, circulatory/breathing). Bivariate relationships (no controlling for confounders) between these 3 factors and four forms of PA guideline achievement showed significant effects of achieving the PA guidelines against various groups of HCs, where more strenuous PA was associated with significantly less HCs in a step-ladder pattern. Multiple regression analyses (controlling for confounders) showed that achievement of PA guidelines was significantly independently associated with self-reported HCs scores in most cases. Psychological HCs were negatively associated with achieving any type of PA; pains/aches were negatively associated with achieving two types of PA or with achieving MSPA guidelines; and circulatory/breathing HCs were negatively associated with achieving the VPA guidelines only. This is the first study in Finland to examine such relationships, and highlights the critical role of PA for the health of these young adults. Programs and policies to strengthen and improve the PA of university students would be beneficial, recognizing the benefits of instilling life-long PA habits among this group of young adults.

## 1. Introduction

University students face many challenges whilst trying to achieve academic success despite financial constraints, personal expectations, peer competition, having to attain good grades or fear of failing/repeating their course [1]. Unsurprisingly, although university students are young healthy adults of high educational level, they report high rates of functional somatic syndromes and physical health complaints (HCs) [2,3,4]. Moreover, despite their reported health needs, university students are reluctant to seek help [5,6,7]. Students might be overcome by their university experience to a magnitude that their physical and mental health could be negatively affected [8].

University students report a range of HCs. In terms of pains and aches, musculoskeletal pain was frequent among university students [9,10,11]. Across university students in the Netherlands, most participants indicated regular or almost continuous discomfort due to their complaints of arm, neck and/or shoulder that have not been caused by a trauma or systemic disease [12]. University students across eight European countries had a 35–43% prevalence of back pain and 43–46% nervousness [13,14]. Likewise, 5.2% and 12.6% of Chinese and German university students reported all three pain/aches (neck/shoulder pain, back/low back pain, headaches) [15]. As for gastrointestinal HCs, university students often reported gastrointestinal complaints [15]; and there was 1.9% prevalence of functional dyspepsia among a Swiss student sample [4]. Likewise, in Germany, irritable bowel syndrome and functional dyspepsia exhibited 6.5% and 4.7% prevalence rates respectively among university students [4]. In terms of mental HCs, in Canada, USA and elsewhere, university students’ psychological distress was significant [16,17,18]. About 17.3% of Chinese and 19% of German university students reported ≥3 psychological symptoms [15]. Among university students in China, the prevalence of general positive psychological symptom was 14.2% [19]; and in Ethiopia, one-third of students had mental distress [20]. University students display high prevalence of mental distress compared to the general population [21].

As regards physical activity (PA), 40–50% of university students are physically inactive [22]. Among university students, 28.4% of the sample were sedentary individuals and 23.6% insufficiently active [23]; and undergraduate students did not engage in adequate PA [24]. Similarly, in the UK, only 51% of the respondents met the recommended levels of moderate to vigorous PA per week, and UK university students were insufficiently active compared with the general population of 16–24 year olds [25]. This is despite that PA and exercise could prevent a range of conditions that include neuromuscular diseases, respiratory, orthopedic, noncommunicable diseases and others [26,27,28,29]; and aerobic exercise could improve and treat primary dysmenorrhea [30].

For university students, a range of variables is associated with HCs and PA. Predictors for physical HCs included gender and age [31,32,33]. Female students more often reported HCs [15]; and the prevalence of psychological symptoms were more in female than male students [19]. Year of study at university is another variable, where research found that the prevalence of irritable bowel syndrome was different for students in earlier vs. later years of study [34]. In the USA, students meeting PA guidelines (vs. not) had lower depression symptomology (*p* = 0.02) [35]; and exercise was associated with improved memory among young adults and reduced depression symptomology [36,37,38]. Living situations may influence PA behaviors, and years of university study, gender, and other features may be associated with university students’ PA behaviors [39,40]. We also explored the relationships between marital status and HCs due to the paucity of research on this relationship.

The literature reveals gaps. Finnish PA research appears to be attentive to adolescents and young children [41,42,43]; population based young adult Finns (aged 18‒29 years) [44]; military conscripts (19‒20 year olds) [45]; or individuals <18 years of age (not reflective of university students) [46]. There is a scarcity of government or country reports committed to the topic despite that PA and physical inactivity are recognized protective and risk factors respectively for a range of chronic conditions. At the population level, a WHO country report found that among Finnish adults (18–65 years), the prevalence reaching the WHO recommended PA levels in 2013 were 32% (males) and 36% (females) [47]. Sparse research on the features of university students’ PA has been implemented in Finland to date, and the very few studies that undertook such investigations employed small samples [48]. One notable exception examined the correlates of PA among a sizeable sample of Finnish university students, but did not report on any HCs [49].

Therefore, the present study bridges these knowledge gaps to appraise the achievement of guidelines four forms of PA: Moderate PA (MPA), vigorous PA (VPA), moderate-to-vigorous PA (MVPA) and muscle strengthening PA (MSPA). The study appraises the relationships of these four forms of PA with a wide range of (20) subjective HCs among a sample of undergraduates at the University of Turku, Finland. The study also simultaneously accounted for five socio-demographic variables (gender, age, year of study, marital status, living arrangements during university terms); and one health variable (self-reported health awareness).

The specific objectives were to:

1. Describe the sample’s characteristics and achievement of guidelines of four forms of PA;
2. Assess the frequency of 21 HCs during the last 12 months, and the level of HCs by gender; and,
3. Assess the relationships between PA guideline achievement and students’ HCs before (bivariate relationships) and after (multivariate relationships) controlling for potential demographic and health behavior confounders.

## 2. Materials and Methods

### 2.1. Sample, Ethics and Procedures

The research and ethics committee at the University of Turku in Turku, Finland, approved the study (Approval # Lausunto 10/2010). A university-wide online survey collected the data using an English language questionnaire (academic year 2013–2014). An email invitation outlining the research aims and objectives was mailed in September 2013 to all (*n* = 4387) undergraduates at all faculties at the University, inviting them to participate in the survey. Students were enrolled at all seven faculties of the University of Turku (Humanities, Mathematics and Natural Sciences, Medicine, Law, Social Sciences, Education and Economics). A pilot survey was undertaken first (May 2013, random sample, 200 students) stratified by faculties. As University students in Finland are fluent in English, the English questionnaire was used for the survey and there was no need for the translation of the questionnaire into Finnish. Very few participants reported any comprehensibility difficulties related to the English questionnaire, and the number of missing values related to items that reasonably could be expected to be answered by all respondents was minimal. The main survey was then launched with the unmodified questionnaire (September 2013). The study used universal sampling, where all students were invited to participate (no inclusion/exclusion criteria). Participation was voluntary and anonymous, and data were confidential and protected (anonymous, no identifiers, strictly accessible only to the research team, stored only on one computer, password/s of the computer and of the files were updated and consistently changed every month, no paper copies were available or stored). Students received an information sheet and contact information in case of any queries and were informed that by completing the survey, they agree to participate in the study. Two weeks later, a follow-up reminder email was sent again to the same sample. Once respondents completed the online survey, their submitted responses were automatically saved and sent to the Student Management Office at the University. The total number of responses was 1177 (response rate: 27%). Students’ mean age was about ≈ 23 (SD 5) years and 832 (70.4%) were females.

### 2.2. Questionnaire

The questionnaire collected general self-reported health data: socio-demographic information (gender, age, year of study, marital status, living arrangements during university terms); lifestyle behaviors (various forms of PA) and HCs; the questionnaire has been used and field-tested across many student populations [50,51,52,53,54,55,56].

Age, sex and year of study at university were based on self-reports. Age was used as a continuous variable.

Marital status: What is your marital status? Response options included single, married, or other (please specify), dichotomized into “single” vs. “married or in relationship” [57,58].

Accommodation (living arrangements) during semester time: “Where do you live during university/college term time?”, dichotomized into “living with parents” vs. “not living with parents” [59,60].

Health awareness (one item): students were asked about their general awareness (surveillance) of their health “To what extent do you keep an eye on your health?” (4-point response: 1 = “not at all”, and 4 = “very much”) [52].

Assessment of self-reported health complaints (22 items): Students were asked how often they have had health complaints (subjective reports of physical or psychosomatic symptoms or discomfort) in the last year. Responses were coded in a four-point scale from never to very often. The following symptoms were asked about: depressive mode, nervousness/anxiety, mood swings, difficulties to concentrate, fear/phobia, sleep disorders/insomnia, nightmares, fatigue, lack of appetite, stomach trouble/heartburn, abdominal problems, neck and shoulder pain, back pain, diarrhea, constipation, headaches, trembling hands, trembling, rapid heartbeat/circulatory problems, breathing difficulties, speech impediment and weight gain/weight loss [1,57]. The last HC (weight gain/weight loss) was dropped from further analysis, because of unclear precision. Given the results from the factor analysis, three components were developed with nine variables for psychosomatic complaints (Cronbach’s alpha = 0.858), seven variables for pain and aches (Cronbach’s alpha = 0.735) and, finally, five variables for circulatory/breathing symptoms (Cronbach’s alpha = 0.736). A health complaint score was constructed for each of the three components (detailed in the statistical analysis section below).

#### PA Variables

MPA (1 item): “On how many of the past 7 days did you participate in moderate exercise for at least 30 min (e.g., biking slower than 10 miles per hour, brisk walking, water aerobics, gardening, tennis (doubles) or dancing (social))?” Participants responded with 0–7 days. We employed a cut-off of ≥5 days/week [61].

VPA (1 item): “On how many of the past 7 days did you participate in vigorous exercise for at least 20 min (exercise for at least 20 min that made you sweat or breathe hard, such as basketball, jogging, fast dancing, swimming laps, jumping rope, tennis (singles), fast bicycling or similar aerobic activities)?” Students responded with 0–7 days. We employed a ≥3 days/week cut-off, in line with the American Heart Association guidelines [61].

MVPA (1 item): Was computed by combining together moderate PA and vigorous PA. All students who achieved either moderate or vigorous PA at the recommended level were designated as achieving MVPA (AHA guidelines for vigorous PA) [61].

MSPA (1 item): “On how many of the past 7 days did you do exercises to strengthen or tone your muscles (such as toe touching, knee bending, leg stretching, or push-ups, sit-ups or weight lifting)?” Participants answered 0–7 days. We used the cut-off of ≥2 days/week [61].

### 2.3. Statistical Analysis

Descriptive and inferential statistics characterized the study sample and tested hypotheses. Descriptive results for quantitative variables (e.g., age) were presented as mean ± standard deviation (SD; for normally distributed data), while numbers (percentage) were reported for qualitative variables (e.g., gender) for the whole sample and by gender. Bivariate analysis (Independent sample t-test, Pearson chi-square or Fisher exact test as appropriate) assessed the relationship of sociodemographics (e.g., age, year of study, marital status, accommodation during university terms, health awareness) and four forms of PA guideline achievement (MPA, VPA, MVPA, MSPA) by gender. Assessment of self-reported HCs included 22 items, where respondents was asked how often they have had HCs in the last year for each item on a four-point scale (1 = never, 2 = rarely, 3 = sometimes, and 4 = very often), later recoded into two categories “never or rarely” vs. “sometime or very often” (1 and 2= 0 vs. 3 and 4= 1). Overall prevalence of each HC (sometime or very often) during last 12 months for the whole sample and by gender were reported. Pearson chi-square or Fisher exact test as appropriate compared the proportion of each HC (sometime or very often) during last 12 months by gender. For all the variables under examination, the missing values percentage were ≤1.5% except for one HC (speech impediment) where 56/1177 (4.8%) were missing. The available number and percentage for each variable are reported and we did not use any imputation for missing values.

Factor analysis (Varimax with Kaiser Normalization rotation) reduced the 22 HCs into meaningful main factors. One HC (weight gain/weight loss) was dropped from further analysis, because of unclear precision/low communality value (0.279). In line with similar studies [1,14,52], and to facilitate the interpretation of the findings, three factors with good loadings emerged, namely psychosomatic (9 variables), pain and aches (7 variables) and circulatory/breathing (5 variables) HCs. Cronbach’s Alpha assessed the internal consistency (reliability analyses) of the items that comprised each of the three factors. The HCs score for each factor (psychosomatic complaints score, pain/aches score, circulatory/breathing HC score) was constructed as a sum of the responses to the questions that comprised it. Higher values correspond to more severity i.e., more perceived psychological (range 9–35), pains/aches (range 7–27) and circulatory/breathing (range 5–18) HCs score respectively. Overall self-reported HCs mean score (last 12 months) for each HCs factor was reported and compared by gender using Independent sample t-test. Similar statistics was also used to assess the relationship between the average score for each HCs factor and PA achievement (MPA guideline achieved vs. not achieved; VPA guideline achieved vs. not achieved; MVPA guideline achieved vs. not achieved; and MSPA guideline achieved vs. not achieved). One Way ANOVA was used to compare the overall self-reported health complaints mean score (last 12 months) for each HCs factor among the four forms of PA achievement (None achieved; MPA achieved only; VPA achieved only; Both MPA and VPA achieved). Post hoc multiple comparisons test was performed for all pair wise comparisons.

Multiple linear regression assessed the associations between each of the four forms of PA guideline achievement (MPA, VPA, MVPA and MSPA) and each of the three different HCs scores, adjusting for potential confounders (age, sex, year of study, marital status, accommodation during semesters, health awareness). Standardized beta-coefficients are additionally presented to allow comparison of results between the three different HCs scores. Model assumption were assessed graphically for multivariate normality (residuals plots were normally distributed); and homoscedasticity (plot of standardized residuals versus predicted values that variance of error terms are similar across the values of the independent variables). Model assumptions were fulfilled for the psychological HC score and the pains/aches HC score, but not for the circulatory/breathing HC score (residuals were not normally distributed). Hence, the results of the multiple linear regression analysis of circulatory/breathing HCs should be interpreted with caution. A “*p*” value < 0.05 (two-tailed) was considered statistically significant. All statistical analyses were performed using Statistical Package for Social Sciences Version 24 (SPSS).

## 3. Results

### 3.1. General and PA Characteristics of the Sample

The majority of respondents either attended Technology and Science (n = 328, 28.5%) or Humanities disciplines (n = 327, 28.5%), while remaining were from faculty of Education and law (n = 188, 16.4%), Economics (n = 138, 12%), and Medicine (n = 168, 14.6%). Table 1 shows the sociodemographic features and four types of PA achievement for the whole sample and by gender. There were more females (70.4%), mean age was 23 years, and about half the sample (47.1%) were first year students. Respondents who were married or in relationship comprised 50.6%, with slight but significant differences by gender. A total of 66.6% of participants were not living with their parents during semester time, with no gender differences. The majority of the students (86.3%) had high health awareness, with significantly higher proportions of females than males expressing that they kept an eye on their health to some extent/very much. Generally low proportions of students achieved the international guidelines for MPA (16.9%) and VPA (29%), and slightly more than a third of the sample achieved the MVPA international guidelines, with no gender differences. However, for MSPA, 41.4% of students achieved the international guidelines, with significantly higher proportions of males than females achieving this recommendation.

### 3.2. Factor Analysis of 21 Self-Reported Health Complaints

Table 2 depicts the results of the factor analysis for HCs for the Finnish sample. The HCs fitted adequately into a three-factor solution, namely: psychological (9 items); pains/aches (7 items); and circulatory/breathing (5 items). For most variables, loading was >0.53, with only two variables loading at 0.463 (lack of appetite) and 0.419 (headaches).

### 3.3. Prevalence and Number of Health Complaints in Last 12 Months

Table 3 shows that the whole sample, fatigue was the most reported HC (60.4%) followed by neck and shoulder pain (59.8%) and headaches (46.6%). For males, fatigue was the most reported HC (42%) followed by neck and shoulder pain (37.2%) and difficulties to concentrate (35.4%). For females, neck and shoulder pain was the most reported HC (69.2%) followed by fatigue (68.2%) and headaches (53.8%). There were gender differences across the majority of HC, where higher proportions of females reported HC than their male counterparts.

### 3.4. Self-Reported Health Complaints Mean Score by Gender

Table 4 shows that females had persistently and significantly higher perceived HCs mean scores than males during the last 12 months across the three groups of HCs.

### 3.5. Bivariate Relationships Between Achievement of Physical activity guidelines and Health Complaints

In terms of the bivariate relationships, Table 5 depicts that achieving any of the various forms of PA and muscles strengthening guidelines was consistently and significantly associated with a lower less HCs score (i.e., less health complaints). This relationship was observed in all instances except for the relationship between achieving the MPA guidelines and circulatory/breathing score.

Table 6 shows that the bivariate relationships between achieving the various forms of PA and HCs exhibited a consistent significant descending gradient relationship (the more PA guidelines achieved, the less HCs reported). For instance, achieving both MPA and VPA guidelines was associated with less HCs than achieving the VPA guidelines, which in turn was associated with less HCs than achieving the MPA guidelines. This was true across the three groups of HCs.

**Figure 1:**
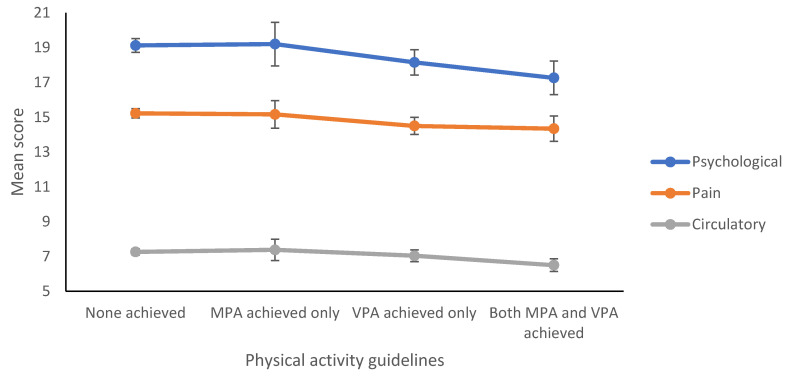
Bivariate Relationships between Physical Activity Guidelines Achievement and Health Complaints Among Undergraduates, University of Turku, Finland. (Circle represents the mean score, error bars are 95% CIs for the mean).

Figure 1 shows the bivariate relationships between PA guidelines achievement and HCs score among the current Finnish undergraduates. It depicts the negative trend (downward slope) that represents the decrease in HCs as more strenuous PA guidelines are achieved, and is consistent for each of the three HCs scores.

### 3.6. Achievement of Physical Activity Guidelines as Independent Factors Associated with Self-Reported Health Complaints

Table 7 shows that achieving each of the MPA, VPA, MVPA or MSPA guidelines was a significant independent negative predictor of psychological HCs. Likewise, achieving each of the VPA, MVPA or MSPA guidelines was a significant independent negative predictor of pains/aches HCs. In addition, achieving the VPA guidelines was a significant independent negative predictor of circulatory/breathing HCs. The psychological HCs were negatively associated with achieving any type of PA; the pains/aches HCs were negatively associated with achieving two types of PA or with achieving MSPA guidelines. Finally, the circulatory/breathing HCs were negatively associated with achieving the VPA guidelines only.

## 4. Discussion

Very little research on the features of various forms of PA of university students and its relationship with HCs has been conducted in Finland to date. The current study described the sample’s characteristics and achievement of guidelines of four forms of PA; assessed the prevalence of 21 HCs during the last 12 months, and factor analyzed them into three groups of HCs; and, appraised the associations between the frequency of the HCs and achievement of the international guidelines of various forms of PA, adjusting for students’ demographic characteristics and health behaviors. The main findings were that achievement of the international guidelines of various forms of PA (MPA, VPA, MVPA, MSPA) guidelines was a significant independent negative predictor for a range of HCs, after controlling for a variety of sociodemographic and health variables.

Our factor analysis generated a three-factor solution: psychological; pains/aches; and, circulatory/breathing groups of HCs, in agreement with other research [1]. An inquiry among undergraduates in the UK and in Egypt employed the same questionnaire as the current study, but reported a four-factor solution for the HCs: Three groups as in the current study, and in addition, a gastrointestinal group of HCs [52]. It is difficult to speculate why we observed a three (not four) factor solution in Finland, particularly that the rates of the three gastrointestinal HCs (diarrhea, constipation, abdominal problems) reported in the UK and Egypt [52] were not very significantly different that those reported in Finland. Another study of university undergraduates in Libya employed the same questionnaire as the current study, but did not factor analyze the HCs into components as undertaken in the current research; rather, the Libyan study used only the three most prevalent health complaints in their analysis [57]. In contrast, we preferred to undertake factor analysis for the 21 HCs and group them into harmonious groups, and then assessed the relationships between these groups of HCs and the various forms of PA. Such analysis that mobilizes all the HCs under examination would be more representative of the data and utilizes the information inherent in all the reported HCs rather than using the most prevalent three HCs only whilst paying less attention to the remaining HCs.

In terms of the prevalence of self-reported HCs during last 12 months, the four most reported HCs in the current sample were fatigue (60.4%), neck and shoulder pain (59.8%), headaches (46.6%) and back pain (45.8%). These HCs are in agreement with findings of university undergraduates in the UK and Egypt (same questionnaire used as in current study), where the HCs reported frequently across these two samples included fatigue and headaches, as well as back pain in the UK [52]. Likewise, among university students in Libya (same questionnaire used as in current study), headaches (60.6%) was one reported of the four symptoms that most often occurred sometimes/very often in the last year [57]. The prevalence of back pain (45.8%) observed in the current study also supports findings of university students across eight European countries who reported a 35%–43% prevalence of back pain [13,14].

As for psychological HCs, the prevalence of nervousness (42.3%) in this current sample is in support of university students in eight European countries (43%-46% prevalence of nervousness) [13,14]. Likewise, about one third of the current Finnish students reported depressive mood, supporting others where more than half of the university students reported depression since the beginning of university [62]. Generally, with the exception of two HCs (speech impediment and trembling), our sample exhibited HCs ranging from 10.2% (breathing difficulties) to 60.4% (fatigue), in line with other research where high prevalence rates for poor physical health have similarly been reported among university and university students [63].

In terms of levels of PA guideline achievement, the current sample demonstrated low levels of achievement (MPA 16.9%, VPA 29%, MVPA 36.1%, MSPA 41.4%). Generally, low levels PA guideline achievement among university students are observed globally: in the USA, 41.9% met public health MVPA recommendations [64]; and in the UK, 12.4%, 33.1% and 23.9% met the MPA, VPA and MSPA guidelines respectively [56]. It is not absolutely clear whether university students generally are fully aware of the PA guidelines and recommendations, as among university students in the USA, accurate knowledge of the PA recommendations was very low [65]. The PA guidelines achievement levels we observed among these young Finns were all <50%, which is concerning, given the importance of PA or the health/wellbeing of these young adults. Coordinated strategies and intervention programs are required to seek these Finnish undergraduates in order to assess whether they are actually aware of the guidelines, to encourage active lifestyles and stimulate more PA. This is important, particularly that university students who were aware of the recommendations were in later stages of PA behavior, and were also significantly more physically active than those who were not aware of the recommendations [66].

As for PA, the bivariate relationships between PA and HCs (before controlling for confounders under examination) showed that achieving the guidelines of most types of PA was negative predictor against various groups of HCs among this Finnish sample (Table 5). In addition, for such bivariate relationships, Table 6 and Figure 1 exhibited an explicit, consistent and significant relationship between PA and HCs, where achieving the guidelines of more forceful (strenuous or robust) forms of PA was associated with significantly less HCs in a harmonious step-ladder manner. This observation was true for the three different groups of HCs across our sample, suggesting that the more PA guidelines achieved, the more protection against HCs is conferred. Such findings agree with others where our findings of the positive effects of PA against HCs agree with other research among different populations. For adolescents in the USA and Canada, positive health indicators were uniformly positively related to PA, while two negative health indicators were negatively related to PA [67]; and among Portuguese adolescents, PA (times/week) was negatively associated with reports of feeling nervous among girls, and with headaches, feeling low, irritability, and feeling nervous among boys [68]. Likewise, at the population level, a wide-ranging review of the possibility of exercise as a stress-buffer found that about 50% of the studies reported at least partly supportive results that individuals with high exercise levels exhibit less health problems when they confront stress [69]. Indeed, PA is essential to normal development and health among the youth [70,71], and university students’ beliefs about PA were associated with changes in their daily exercise following exposure to naturally-occurring stressors, where such beliefs might be responsive to intervention programs that encourage adaptive coping with stress and enhanced PA [72]. Nevertheless, some studies did not observe an association between exercise and cardiorespiratory fitness on, e.g., subjective memory complaints among young adult university students [35].

As for the multiple regression analysis (Table 7), controlling for a range of variables that could act as confounders in the PA-HCs relationship (age, sex, year of study, marital status, accommodation during semesters and self-reported health awareness), we found that the relationships observed in the bivariate analysis still held true in most cases even after such controlling for confounding was undertaken in the multiple regression analysis. The achievement of four PA guidelines significantly and independently predicted self-reported HCs scores among this sample of Finnish undergraduates in the majority of cases. The psychological HCs were negatively associated with achieving any type of PA; pains/aches were negatively associated with achieving two types of PA or with achieving MSPA guidelines; and circulatory/breathing HCs were negatively associated with achieving the VPA guidelines only.

Such regression findings represent strong evidence of the “net” positive effects of PA on HCs. Our findings agree with others where, among undergraduate university students, hierarchical regression showed that strenuous PA participation predicted the number of health complaints [73]; and in the UK, students with higher PA reported better outcomes for mental and personal well-being [25]. Among university students in China, after adjusting for sociodemographic characteristics, physical health and physical activity, binary logistic regression analysis showed that the total sedentary time were significantly associated with depression [74]. Similarly, among school-aged adolescents, PA level was independently associated with psychological HCs, where the average psychological HCs score increased when moving from the high to low PA categories [75]. Likewise, for middle age and older adults, after controlling for covariates, subjective memory complaints were strongly related to PA, where participants with more PA had lower frequency of memory complaints [76,77]. In agreement, in China, among women aged 20–40 years, PA was related to reduced fatigue and nervousness [78]; and in Germany, a longitudinal inquiry of the development of PA, physical fitness, and subjective physical complaints found that high initial levels of PA and fitness protected against high subjective physical complaints, and that an increase of fitness resulted in a decrease of subjective physical complaints [79]. Similarly, among middle-aged Americans, moderate levels of household PA and leisure-time PA were significantly oppositely linked to subjective memory complaints [76].

The study has limitations. The study is cross sectional: No cause–effect relationships can be deduced. Females were slightly over-represented, although this is a reality at higher education institutions in most countries, hence our regression models were adjusted for gender. Selection bias, where potential respondents with HCs might have been less likely to attend at university during the data collection or participate in the survey is possible. Self-reports assessed the HCs (no objective validation undertaken). However, no valid external evaluation of health complaints exists since doctors’ ratings are also subject to a great extent on patients’ descriptions. Online surveys usually have low response rates, and our response rate was low (27%), hence generalizations need to be very cautious, although our response rate was higher than the response rates of many online surveys e.g., 11.6% [80], 14% [81], or 25% [82]. Although the survey was administered well into the university academic year, respondents were asked about how often they have had HCs during the last year, where some first-year students could have misinterpreted the question to be asking about their high school year rather than their current university year. Ethnicity data was not available; this could have been beneficial to explore whether HCs, PA and their relationships were uniform across various ethnic groups. In the factor analysis, the names given to the factors were kept as representative and simple as possible; nevertheless, the “pains and aches” component incorporated gastrointestinal problems, whereas the “circulatory and breathing” component included trembling. The findings of the current study were sometimes compared with similar findings from countries that have different cultural, educational, socio-demographic context, e.g., Egypt or the UK. Nevertheless, when similarities in findings exist in terms of the current findings with those of other countries, then such “parallelism” serves to strengthen the findings in terms of associations between the variables which exist in two totally unrelated countries. Future research could address these limitations. Despite this, the study has many strengths. The large sample size facilitated the computation of precise estimates across students at different faculties and studying different disciplines. The study operationalized a variety of socio-demographic characteristics and health behavior variables as well as the achievement of four different forms of PA. We also assessed the relationships between marital status and HCs thus contributing information to the very sparse research on this relationship. To the best of our knowledge, this is the first study among university students in Finland that examined in detail, the HCs, and their associations with achievement of guidelines of four different forms of PA, whilst simultaneously accounting for a range of other potential confounders.

## 5. Conclusions

Among this sample of Finnish undergraduates at one university, the high levels of HCs and the low levels of achievement of PA guidelines are concerning. High rates of HCs were generally observed, particularly fatigue, neck and shoulder pain, headaches, and back pain. Low rates of PA guidelines achievement were observed for MPA, VPA, MVPA, and MSPA. Bivariate relationships between PA and HCs (before controlling for confounders) showed significant relationships of achieving the guidelines of most types of PA against various groups of HCs, where achieving the guidelines of more forceful (strenuous or robust) PA was associated with significantly less HCs in a step-ladder pattern. After controlling for potential confounders, the achievement of the four PA guidelines independently predicted self-reported HCs scores among this sample of Finnish young adults in the majority of cases. Psychological HCs were negatively associated with achieving any type of PA; pains/aches were negatively associated with achieving two types of PA or with achieving MSPA guidelines; and circulatory/breathing HCs were negatively associated with achieving the VPA guidelines only. These findings highlight the critical role of PA in health of the young adults. Programs and policies to strengthen and improve the PA of university students would be beneficial, recognizing the benefits of instilling life-long PA habits among this group of young adults.

## Figures and Tables

**Table 1:** Socio-demographic and physical activity achievement by gender among undergraduates, University of Turku, Finland (N = 1177).

Variable	Total 1177	Male 346 (29.6%)	Female 823 (70.4%)	*p* Value *
	N (%)	n (%)	n (%)	
Age years *^a^* (M, SD)	22.96 (5.21)	22.83 (4.35)	23.0 (5.55)	0.58
Year of Study				0.017
1st	549 (47.1)	178 (51.7)	371 (45.2)	
2nd	343 (29.4)	106 (30.8)	237(28.9)	
3rd	250 (21.5)	54 (15.7)	196 (23.9)	
≥4th	23(2.0)	6 (1.7)	17 (2.1)	
Marital status				0.001
Married or in relationship	588 (50.6)	148 (42.8)	440 (53.9)	
Single	575 (49.4)	198 (57.2)	377 (46.1)	
Accommodation during semester				0.417
With parents	389 (33.4)	109 (31.7)	280 (34.1)	
Not with parents	775 (66.6)	235 (68.3)	540 (65.9)	
Health awareness				<0.001
Not at all/not much	159 (13.7)	70 (20.4)	89 (10.9)	
To some extent/very much	1001 (86.3)	273 (79.6)	728 (89.1)	
Physical activity achievement				
MPA guidelines	196 (16.9)	54 (15.7)	142 (17.4)	0.492
VPA guidelines	334 (29.0)	110 (32.3)	224 (27.6)	0.110
MVPA guidelines	416 (36.1)	127 (37.2)	289 (35.6)	0.604
MSPA guidelines	480 (41.4)	186 (54.1)	294 (36.0)	<0.001

MPA: moderate PA; VPA vigorous PA; MVPA moderate-to-vigorous PA; MSPA: muscle strengthening PA; Numbers in parenthesis represent column percentages unless otherwise indicated; * Two-sided *p*-values based on Pearson chi square and Fisher exact test for categorical variables, and Student t test for continuous scale variables for comparison between means; *^a^* mean (standard deviation); numbers might not sum up to total because of missing values.

**Table 2:** Factor analysis and loading using varimax rotation of 21 self-reported health complaints into three components.

Health Complaint	Component		
	Psychological (9 Items)	Pains/Aches (7 Items)	Circulatory/Breathing (5 Items)
Cronbach’s alpha	0.858	0.735	0.736
Eigenvalue	6.868	1.536	1.278
% of Variance	32.703	7.313	6.088
Depressive mood	0.749		
Nervousness/anxiety	0.699		
Mood swings	0.719		
Difficulties to concentrate	0.649		
Fear/phobia	0.572		
Sleep disorders/insomnia	0.594		
Nightmares	0.564		
Fatigue	0.536		
Lack of appetite	0.463		
Stomach trouble/heartburn		0.685	
Abdominal problems		0.602	
Neck and shoulder pain		0.601	
Back pain		0.542	
Diarrhea		0.591	
Constipation		0.543	
Headaches		0.419	
Trembling hands			0.730
Trembling			0.759
Rapid heartbeat/circulatory problems			0.545
Breathing difficulties			0.533
Speech impediment			0.593

Rotation Method: Varimax with Kaiser Normalization; Kaiser–Meyer–Olkin Measure of Sampling Adequacy = 0.926; Bartlett’s Test of Sphericity (Chi-square test = 7102.89, df = 210, *p*-value < 0.001).

**Table 3:** Prevalence of self-reported health complaints during last 12 months by gender among undergraduates, University of Turku, Finland.

Health Complaint	Total (*n* = 1177)	Male 346 (29.6%)	Female 823 (70.4%)	*p* Value*
	Sometimes/ Very Often n (%)	Sometimes/ Very Often n (%)	Sometimes/ Very Often n (%)	
**Psychological**				
Depressive mood	387 (33.3)	89 (25.8)	298 (36.4)	<0.001
Nervousness/anxiety	493 (42.3)	98 (28.5)	395 (48.1)	<0.001
Mood swings	429 (36.8)	62 (18)	367 (44.7)	<0.001
Difficulties to concentrate	529 (45.4)	122 (35.4)	407 (49.7)	<0.001
Fear/phobia	159 (13.7)	35 (10.2)	124 (15.1)	0.025
Sleep disorders/insomnia	390 (33.5)	92 (26.8)	298 (36.3)	0.002
Nightmares	264 (22.7)	42 (12.2)	222 (27.1)	<0.001
Fatigue	701 (60.4)	145 (42)	556 (68.2)	<0.001
Lack of appetite	157 (16.1)	25 (7.3)	132 (13.5)	<0.001
**Pains/aches**				
Stomach trouble/heartburn	494 (42.5)	88 (25.7)	406 (49.5)	<0.001
Abdominal problems	225 (19.3)	31 (9)	194 (23.7)	<0.001
Neck and shoulder pain	695 (59.8)	128 (37.2)	567 (69.2)	<0.001
Back pain	534 (45.8)	110 (31.9)	424 (51.6)	<0.001
Diarrhea	212 (18.2)	48 (14)	164 (20)	0.015
Constipation	134 (11.6)	20 (5.8)	114 (14.1)	<0.001
Headaches	544 (46.6)	101 (29.3)	443 (53.8)	<0.001
**Circulatory/breathing**				
Trembling hands	137 (11.8)	45 (13.2)	92 (11.2)	0.329
Trembling	55 (4.8)	11 (3.2)	44 (5.4)	0.105
Rapid heartbeat/circulatory problems	245 (21.1)	35 (10.2)	210 (25.6)	<0.001
Breathing difficulties	119 (10.2)	20 (5.8)	99 (12)	0.001
Speech impediment	35 (3.1)	12 (3.6)	23 (3)	0.575

All percentages are row percentages rounded to one decimal point; Bolded cells indicate some of the higher frequency symptoms/health complaints during last 12 months. Numbers might not sum up to total because of missing values. * Two-sided *p*-values based on Pearson chi square or Fisher exact test.

**Table 4:** Self-reported health complaints mean score (last 12 months) by gender among undergraduates, University of Turku, Finland.

Health Complaint	Total (*n* = 1177)	Male 346 (29.6%)	Female 823 (70.4%)	*p* Value*
	M (SD)	M (SD)	M (SD)	
Psychological score ^a^	18.76 (5.45)	16.46 (5.25)	19.73 (5.25)	<0.001
Pains/aches score ^b^	14.98 (3.72)	12.98 (3.36)	15.83 (3.53)	<0.001
Circulatory/breathing score ^c^	7.14 (2.37)	6.75 (2.21)	7.31 (2.42)	<0.001

M: mean; SD: standard deviation; ^a^ range: 9–35, higher values correspond to more perceived psychological symptoms; ^b^ range: 7–27, higher values correspond to more perceived pains/aches symptoms; ^c^ range: 5–18, higher values correspond to more perceived cardiovascular/breathing symptoms. * Two-sided *p*-values based on Student t test for comparison of means between males and females for each of the health complaint factors.

**Table 5:** Bivariate relationships between four types of physical activity guidelines achievement and health complaints among undergraduates, University of Turku, Finland.

Physical Activity Achievement	Health Complaint Score					
	Psychological		Pains/Aches		Circulatory/ Breathing	
	M ± SD	*p* Value	M ± SD	*p* Value	M ± SD	*p* Value
MPA guidelines		0.046		0.217		0.073
Not achieved	18.92 ± 5.43		15.06 ± 3.70		7.21 ± 2.38	
Achieved	18.06 ± 5.50		14.69 ± 3.78		6.86 ± 2.33	
VPA guidelines		<0.001		0.002		0.010
Not achieved	19.12 ± 5.45		15.21 ± 3.68		7.26 ± 2.41	
Achieved	17.84 ± 5.37		14.45 ± 3.72		6.85 ± 2.27	
MVPA guidelines		0.003		0.006		0.044
Not achieved	19.12 ± 5.43		15.22 ± 3.69		7.26 ± 2.37	
Achieved*	18.10 ± 5.45		14.59 ± 3.70		6.95 ± 2.37	
MSPA guidelines		<0.001		<0.001		0.010
Not achieved	19.42 ± 5.43		15.45 ± 3.69		7.32 ± 2.44	
Achieved	17.89 ± 5.37		14.39 ± 3.67		6.94 ± 2.26	

MPA: moderate PA; VPA: vigorous PA; MVPA: moderate-to-vigorous PA; MSPA: muscle strengthening PA; * Achieved either MPA or VPA, or both.

**Table 6:** Bivariate relationships between three (mutually exclusive) categories of physical activity guidelines achievement and health complaints among undergraduates at the University of Turku, Finland.

	Health Complaint Score					
	Psychological		Pains/Aches		Circulatory/ Breathing	
	(M, SD)	*p* Value	(M, SD)	*p* Value	(M, SD)	*p* Value
Physical activity guidelines		0.002		0.018		0.013
None achieved	(19.12, 5.43) *		(15.22, 3.69)		(7.26, 2.37) *	
MPA achieved only	(19.20, 5.67)		(15.16, 3.61)		(7.38, 2.74)	
VPA achieved only	(18.15, 5.41)		(14.50, 3.64)		(7.04, 2.41)	
Both MPA and VPA achieved	(17.26, 5.26) *		(14.34, 3.87)		(6.50, 1.94) *	

MPA: moderate PA; VPA: vigorous PA; MVPA: moderate-to-vigorous PA; MSPA: muscle strengthening PA. * Both MPA and VPA achieved were significantly different from none achieved using post hoc multiple comparisons test.

**Table 7:** Achievement of four physical activity guidelines as independent factors associated with self-reported health complaint scores among undergraduates, University of Turku, Finland *.

Physical Activity Achievement	Health Complaint Score								
	Psychological			Pains/Aches			Circulatory/Breathing		
	Std-β	β (95% CI)	Adj R^2^ (β)	Std-β	β (95% CI)	Adj R^2^ (β)	Std-β	β (95% CI)	Adj R^2^ (β)
MPA achieved	**−0.059**	**−0.851 (−1.661; −0.041) *p* = 0.040**	**0.085**	−0.046	−0.452 (−0.994;0.090) *p* = 0.102	0.122	−0.054	−0.341 (−0.714; 0.032) *p* = 0.073	0.017
VPA achieved	**−0.085**	**−1.025 (−1.704; −0.347) *p* = 0.003**	**0.089**	−0.081	**−0.661 (−1.115; −0.208) *p* = 0.004**	**0.127**	**−0.070**	**−0.365 (−0.677; −0.052)** ***p* = 0.022**	**0.019**
MVPA achieved	**−0.077**	**−0.869 (−1.508; −0.230) *p* = 0.008**	**0.088**	−0.080	**−0.618 (−1.045; −0.191) *p* = 0.005**	**0.127**	−0.056	−0.274 (−0.568; 0.020) *p* = 0.068	0.018
MSPA achieved	**−0.085**	**−0.940 (−1.582; −0.299) *p* = 0.004**	**0.089**	−0.090	**−0.681 (−1.109; −0.252) *p* = 0.002**	**0.128**	−0.059	−0.285 (−0.581; 0.010) *p* = 0.058	0.018

* Multiple linear regression; MPA: moderate PA; VPA: vigorous PA; MVPA: moderate-to-vigorous PA; MSPA: muscle strengthening PA; Std-ß: standardized beta coefficient; ß: beta coefficient; CI: confidence interval; Adj: Adjusted (models adjusted for age, sex, year of study, marital status, living with partner, and self-reported health awareness); Bolded cells indicate statistical significance (*p* < 0.05). Interaction between gender and the four forms of PA for each HCs score model were examined and no significant interaction effects were found.
